# Licensing and regulation of the mental health residential care facilities: A case study from the Sedibeng district, South Africa

**DOI:** 10.1371/journal.pmen.0000308

**Published:** 2025-05-07

**Authors:** Samantha Mhlanga, Frances Griffiths, Lesley Robertson, Jane Goudge

**Affiliations:** 1 Centre for Health Policy, Faculty of Health Sciences, University of Witwatersrand, Johannesburg, South Africa; 2 Medical School, University of Warwick, Warwick, United Kingdom; 3 Department of Psychiatry, University of Witwatersrand, Johannesburg, South Africa; 4 Community Psychiatry, Sedibeng District Health Services, Sedibeng, South Africa; PLOS: Public Library of Science, UNITED KINGDOM OF GREAT BRITAIN AND NORTHERN IRELAND

## Abstract

The World Health Organization advocates for the regulating of mental health accommodation services to ensure that they use a person-centered approach and promote human rights. However, many low-middle-income countries still face challenges that prevent them from delivering services according to such regulations. In this study, we explore the experiences of regulating mental health residential facilities in one district of South Africa. We conducted a qualitative study using in-depth interviews and observations from October 2022 to June 2023. Our participants were district governance and compliance team members, and managers, staff, residents and their families of three residential facilities. The governance team tried to ensure facilities complied with regulatory standards, assisting them where possible to meet those standards. Managers and family members understood that enforcing regulation will help improve service delivery. However, managers reported that some regulatory standards were more appropriate for hospitals than residential facilities. Facility staff felt neglected and undervalued by the governance team. Residents found the inspection process intrusive. Managers reported that their experience of providing residential services was undervalued in the regulatory process and that the funding for their facilities was insufficient for them to meet current standards. The governance team were aware there was a shortage of residential care facilities so had to decide which facilities to license even when they did not meet current standards. Current inspection, licensing and regulation of mental health residential facilities in South Africa is not yet fit for purpose and may hinder residents to achieve independency and quality of life. Involvement of service users and providers in mental health care planning may help align the licensing regulations to residential care services that are truly community-based.

## Summary

Why was this study done?

What did researchers do and find?

What do these findings mean?

- In response to Life Esidimeni tragedy, the Minister of Health gazetted the national policy guidelines for licensing of residential care and/or day care centres.
- The Gauteng Department of Health is responsible for licensing the residential facilities and the NGO governance and compliance teams (NGCT) were established to support and monitor residential facilities to be licensed.
- There is no evidence about the experiences of the licensing and regulating process for mental health residential facilities.
- We used a qualitative approach, and interviewed the NGCT, residential facility managers, staff, residents and their family members.
- The facility managers felt they were not consulted during implementation of the regulations.
- The facility managers raised concerns about the hospital like requirements for the licensing.
• The dual role of supporting and inspecting the residential facilities created tension for the NGCT.
- There is a need to clarify the purpose of residential facilities, whether they should be considered as health establishments or social care facilities.
- The NGCT should be trained so that they are able to balance their dual role of inspecting and supporting the residential facilities.
- Service users and providers of mental health residential facilities should be involved in decision making to improve the quality of the rehabilitative environment.

## Introduction

Mental health residential facilities enable people living with serious mental illness (PLSMI) to live in society rather than psychiatric hospitals [1,2]. In general, they provide a home, food, personal care, access to health care and psychosocial rehabilitation [3]. The World Health Organization (WHO) emphasises the importance of regulating residential facilities to safeguard the rights of PLSMI and ensure that the services meet standards of quality and safety [2].

In England and Wales, the Care Quality Commission, which has been regulating the standards of health and social care since 2009, has closed down residential facilities that fail to protect residents from the risk of unsafe practices such as medication mismanagement and human rights violations [4]. In Australia, the Commission on Safety and Quality in Health Care introduced national standards for mental health services in 2010 which include the regulation of non-governmental mental health residential facilities [5]. In India and Ghana, mental health authorities are responsible for inspecting and registering mental health residential facilities [6,7].

In South Africa, the National Mental Health Policy Framework and Strategic Plan 2023 – 2030 recognises the need to strengthen residential facilities to deliver mental health care [8]. Such facilities include half-way houses, group homes and facilities providing supported living [8]. These facilities are predominantly run by non-governmental organisations (NGO). In this paper, we explore the experience of the licensing and regulating process for mental health residential facilities in one district of South Africa to gain an understanding of the challenges faced by those involved in the process.

## Background

South Africa’s mental health system spends more money on hospital care compared to community-based care [9]. As a result, community-based care services still face challenges such as lack of funding and resources [9]. Serious problems in the regulation of residential facilities were exposed in the Gauteng province of South Africa in what has become known as ‘the Life Esidimeni tragedy’. In 2015, the Gauteng Department of Health ended its contract with ‘Life Esidimeni’, a private hospital group which provided long-stay hospital care for PLSMI to the province. Over a period of 6 months, 1442 PLSMI were discharged from Life Esidimeni hospitals, many of them to NGO. This rapid deinstitutionalisation resulted in the death of 144 patients [10].

When investigating the incident, the health ombudsman found that some NGOs which had received the discharged patients were unlicensed and had insufficient resources to provide adequate mental and physical health care [11]. The ombudsman recommended that the licensing regulations and procedures be reviewed by the National Department of Health and that new processes developed which safeguarded the rights of PLSMI [11]. In response, national policy guidelines were gazetted in March 2018 by the Minister of Health licensing of residential care and/or day care centres for PLSMI and/or severe or profound intellectual disability [12] [Box 1 summarizes the licensing guidelines].

The licensing guidelines apply to all facilities that provide care, treatment and rehabilitation to five or more PLSMI [12], as stipulated in the regulations to the Mental Health Care Act No 17 of 2002 [13]. NGOs providing accommodation services for people who do not have a serious mental illness are registered with and monitored by the Department of Social Development [14]. However, under the Mental Health Care Act, NGOs providing care for five or more PLSMI must be licensed by the Department of Health in addition to being registered with the Department of Social Development [13]. The licensing guidelines regard residential facilities as ‘health establishments’ (which are defined by the Mental Health Care Act as “institutions, facilities, buildings or places where persons receive care, treatment and rehabilitation such as community health and rehabilitation centres”). In clauses 15.3 and 15.4, the licensing guidelines require PLSMI to be admitted to residential facilities for care, treatment and rehabilitation as voluntary, assisted or involuntary mental health care users under the Mental Health Care Act [12]. The licensing guidelines also require such facilities to comply with norms and standards applicable to health establishments (e.g., to include medical procedure rooms and clinical services) and defines the facility manager as a head of health establishment [12]. However, no qualifications are necessary to be a facility manager, and no training of caregivers is delineated.

In its observations on South Africa, the United Nation Committee on the Rights of Persons with Disability (UNCRPD) expressed concern that the licensing guidelines reflect a medical model of disability [15]. The medical model of disability states that people are disabled because of their physical and mental limitations, and focuses on finding a cure to help people with disability live a normal life [16]. The UNCRPD statement implies that the South African regulations still overlook other important factors for PLSMI, such as full inclusion, community integration and equality [15]. The Committee recommends that the regulations need to align with the human rights model of disability.

**Box 1: A summary of the licensing guidelines for mental health residential facilities**

The guidelines provide a framework for licensing of residential and day care centre in order to facilitate community rather than hospital-based care, treatment and rehabilitation of people living with serious mental illness. The guidelines states that the environment where services are delivered should be safe, therapeutic, less restrictive, and protect the basic human rights of the mental health users. They further state that the services should be culture sensitive, and evidence based.

Mental health facilities should be registered as a non-profit organisation (NPO) with the Department of Social Department (DSD) and licenses as a health establishment by the Provincial Department of Health. The licensing application should be accompanied by.

- Proof of registration with DSD as an NPO from the service level agreement (SLA).
- The SLA is between the applicant and the District Health Services’ manager regarding the clinical services which the district will provide to the residents of the NPO.
- Municipal and environmental health certificates for zoning or rezoning (to specify what you want to use the land for); occupation (meets all building requirements); acceptability (to ensure that the food premise comply with general hygiene regulatory requirements); health (to ensure that the premise comply with the National Environmental Health Norms and standards); water supply clearance, compliance (In terms of electrical requirements including backup generator).
- Health risk management contract, clinical protocols of care, rehabilitation programme, and human resources.

An inspection team that consists of but not limited to a psychiatric nurse, psychiatrist, occupational therapist, social worker, dietician, environmental health practitioner should be established in the District Health Services. The team shall inspect mental health facilities quarterly, and provincial department shall conduct annual audits to monitor compliance according to assessment tool and compliance report. The license may be renewed annually subjected to the audit outcome.

The assessment tool and compliance report consist of 21 elements (with sub-elements) for compliance. The inspection team should use this tool to assess the conditions of the facilities and provide comments. The list includes exterior environment, leadership and governance, staff identity and dress code, staff training, records and filing room, infection prevention and control, toilets and bathrooms, food and meals, bed and laundry rooms and corridors, recreational area and programmes, treatment room, reproductive and preventive health care.

*[Adapted from National Health Care Act DA. Policy Guidelines for The Licensing of Residential And/Or Day Care Facilities For Persons With Mental Illness And/Or Severe Or Profound Intellectual Disability. 2003;76]*

The Gauteng Department of Health (GDOH) depends on NGO run residential facilities to enable PLSMI to live in their communities. The GDOH is responsible for licensing the NGOs and the NGO governance and compliance teams (NGCTs) were established to support and monitor NGOs to be licensed [17]. These multidisciplinary teams include professionals from social work, dietetics, environmental health, finance, nursing and occupational therapy. The current team manager, a psychiatric nurse, was originally appointed by Sedibeng mental health department in 2010 to support NGOs running residential facilities with licensing. In 2014, he was joined by another psychiatric nurse. NGC team was formed in 2018 after the Life Esidimeni tragedy. Most of the members joined from 2021 to 2022 with no or very little experience of working with NGOs that provide residential care.

## Methods

### Ethics statement

The study received ethics clearance from the University of Witwatersrand Human Research Ethics Committee [certificate no M220402] and permission to conduct the research was provided by Sedibeng District Health Services.

### Study approach and design

We employed a qualitative approach [18] using a case study design [19] to collect data through observation and face to face individual interviews, as part of a doctoral research about community-based residential facilities.

### Study setting

We selected the Sedibeng district which is in the Southern part of Gauteng Province as our study site, as we were aware of an active NGCT in the district. Comprising urban and rural areas, Sedibeng district has a population of just over one million people, and is characterised by poor infrastructure and high levels of unemployment, poverty and crime [20]. Sedibeng district currently has 14 NGOs providing residential accommodation for 331 adults with serious mental illness.

### Researcher positionality

SM conducted all the study interviews. SM has a social work background, and her training equipped her with research skills such as recruitment and interviewing vulnerable groups. Her training enabled her to build rapport with the participants and interview them with sensitivity. SM was supervised by the co-authors (J.G, F.G, and L.R). During fieldwork, SM met with the supervisors weekly for guidance and support, and to ensure consistency and rigour during data collection.

LR heads up the Sedibeng district specialist mental health team and is responsible for strengthening mental health services using a public health approach. She helped us design the project and ensure that it is locally. She linked us with the Sedibeng NGCT manager who then introduced us to the rest of the NGCT and the NGOs they are working with. LR was not involved in the recruitment of participants or data collection process. She also did not have access to any audio recordings and transcripts. This was to protect participants’ details and ensure that she did not influence how they respond during the interviews. SM assured all the participants that all audios will be kept in a password protected laptop and identifying details will be removed from transcript.

### Sampling and recruitment

Our case is the Sedibeng District Health Services NGCT along with three NGO run residential facilities licensed by GDOH. We sampled three NGOs to ensure feasibility and to ensure thorough data collection and analysis for each one. The NGOs were selected to include those with differing licensing statuses: one’s license had only been renewed 6 months because the building did not meet the necessary standard; the second one was fully licensed for the year; and the third one’s environmental health certificate had expired, and the manager was struggling to renew it due to financial constraints.

#### Sedibeng NGCT.

In collaboration with the NGCT manager we arranged a meeting with the NGCT to explain the study. We invited all team members for an interview about their work, and the challenges they experience when regulating NGOs.

#### Facility managers and staff.

We invited the manager of each facility for interview about the experience and the challenges they experience with the licensing process. With facility manager’s permission we arranged a meeting with the facility staff to explain about the study. We used stratified random sampling to select the staff for interview based on whether they had received training from, or participated in inspection done, by the NGCT. We interviewed them about their experience with the licensing process.

#### Residents.

With facility manager’s permission we arranged a meeting with each resident accompanied by a staff member. In the meeting, we assessed the resident’s capacity to provide consent. We used an activity to assess residents’ capacity to provide consent where we explained the meaning of providing consent using their preferred language and asked them to explain it using their own words to demonstrate understanding [21]. All residents who gave consent were invited for interview about their experience with the licensing process.

#### Family members of residents.

We asked managers to ask family members to participate in the study. SM contacted family members who agreed. We interviewed them about their views about the regulation of NGOs.

### Data collection

SM collected data from October 2022 to June 2023. We established trustworthiness of the study using different methods of data collection such as interviews, observations and reflective diary*.*

#### Observations and fieldnotes.

We asked each facility manager to let us know when the NGCT is coming to the facility so we could observe the team throughout inspection day. Detailed notes about the teams’ engagement with the manager, staff, and residents, reviewing of files, assessment of the NGO environment and resources were written immediately after observation.

#### Interviews.

The interview guide was informed by the standardised tool for quality assessment of mental health supported accommodation developed by Killaspy and colleagues [22]. This tool is completed by the service manager and describes the quality of the following domains: living and therapeutic environment, treatment and interventions, self-management and autonomy, social interface, human right and recovery-based practice. The interview guides were piloted at an NGO run residential facility not included in the study and subsequently refined. Interviews were audio recorded and conducted in English, Sesotho or Isizulu languages by the lead author (SM). Recruitment of participants continued until data saturation was reached [23].

### Data management and analysis

SM transcribed and where necessary translated the interviews in collaboration with 2 PhD students who can also speak the necessary languages. Transcripts were checked against the audio recording and identifiers removed. We used inductive and deductive thematic analysis as described by Lochmiller [24] to analyse individual interviews. We re-read all the interview transcripts and fieldnotes to familiarize ourselves with the data. We use Nvivo software and Word document to generate initial codes which helped us to organise data into meaning groups. After coding, we grouped codes into themes. During analysis, where necessary, local idiomatic use of English was edited for global readers. JG and FG met with SM weekly during analysis, read transcripts, reviewed editing, and coding and discussed themes. LR, who has a management role in the Sedibeng mental health services, did not have access to raw data and was not involved in data analysis. In order to reduce bias, we used both interviews and observations to collect data. SM also kept a reflective diary to document all her observations and details about the research process. All authors met weekly to ensure that there is transparency and consistency during data collection and analysis.

## Results

### The residential facilities

A total of 104 individual interviews were conducted, comprising 10 NGCT members, 3 facility managers, 38 facility staff, 36 residents and 17 family members of residents. One NGCT member preferred not to participate because she had recently joined the team. Despite asking the NGO staff, residents and families about their experiences of the licensing process, relevant data were not elicited in 72 of the interviews. Our data set for analysis was therefore 33 interviews, 10 NGCT members, 3 facility managers, 10 facility staff, 5 residents and 5 family members of residents. A total of 4 themes were generated from the analysis: Perceived benefits of regulation, suitability of licensing guidelines to residential services, challenges in complying with the guidelines and perceptions of residential staff and residents of the NGCT.

**Box 2: Description of the residential facilities and challenges they faced to get licensed.**

**Facility 1:** Started in 2005 and was licensed for 21 residents. They have 3 different houses in a township, each with 3 bedrooms, a kitchen, a bathroom and a living room. A sleeping room is shared by 2–4 residents. Each house has 2 stay-in caregivers administering medication, cooking and monitoring residents to do activities of daily living. The facility was doing well and fully licensed. However, it is not well resourced due to financial challenges. The adjudication was lenient with them for not having updated acceptability/compliant certificate especially regarding environmental health which expired. They still lack a professional nurse, sickbay, have informal structures for laundry, storeroom and activity room. The team is experiencing challenges with the manager because she defends herself. During their meeting with municipality, they said she was not fully honest about the regulations and municipality certificates. The team was also concerned about the manager not being honest about the activities for the residents.

**Facility 2:** Has been operating since 1996. It is currently licensed for 72 residents but providing care to 86 residents. It is a school-like building located in a commercial area, with 4 dormitories sleeping 18–21 residents each. They have 8 stay-in caregivers, 2 resident cooks and a laundry attendant. They have an onsite nurse, assistant nurse and a part-time social worker. The facility was fully licensed for a year, but they don’t have their health certificate. The NGCT raised concerns that the facility lacks hygiene, smells of urine, and they don’t maintain ablution facilities. The team suspected that the facility might be caring for more residents than what they were licensed for. During announced visits, the team found things like new toothpastes, and new soap, and they suspect that they make residents share toiletries. The facility was not following the rehabilitation programme for residents but through inspection, training from the team they now implementing the programme. The team was concerned about residents that are aggressive and those who lack of family relations.

**Facility 3** Founded in 2001 and was licensed for 40 residents. It is a small farm with standalone buildings including a house each for the founder and the manager of the facility, a house with 16 residents sharing, a cottage with 5 females sharing, different homes for residents living on their own. They have 6 caregivers, 6 cleaners, and part-time nurse working during the day only. This facility was initially licensed Johannesburg District, and later moved to be under Sedibeng District. The facility was struggling in terms of the building structure, paint was peeling off the walls, mould, and the smell of urine. As a result, this facility was licensed for 6 months. Through inspections and recommendations, the facility improved but they were struggling with rezoning, acceptability and health certificates. They have 2 informal structures that the family insisted they want their residents to continue using as their sleeping room. They have a small activity room which makes them focus on few residents for the activity program while others are loitering. The team was concerned about the facility giving residents half of their grant money because residents use the money to buy too many cigarettes, cold-drinks, chips and spices, which is not healthy especially for those with chronic like diabetics and high blood pressure.

All the facilities were operating before the Life Esidimeni tragedy. In facility 1 and 3 residents lived in family size units. Facility 2 was a school-like building with 4 dormitories for sleeping and a central kitchen providing food. Box 2 provides further details about each facility and the challenges they faced in relation to licensing. Boxs 3 and 4 provides a summary of observations of the NGCT doing inspection in facility 1 and 2. We were unable to observe facility 3 being inspected as the annual inspection had occurred just before recruitment.

**Box 3: Observations of the NGCT doing an inspection at facility 1**

A team of 9 members were there from 8am till 4pm. All houses were clean, the staff members had name tags, and caregivers were wearing aprons, hair nets and safety boosts. The residents were more smartly dressed than usual and looked scared of the team. The social worker, finance and team administrators worked in the living room with the facility admin and manager extracting information from care files (e.g., clinic dates and family visits) and facility files (e.g., in-services training, performance appraisal). The team nurse, dietician and occupational therapist (OT) were outside assessing each residents’ BMI, sugar levels, blood pressure, eye sights (e.g., cataracts) and teeth. Later they used residents’ sleeping room to interview residents about their stay at the facility and activities they do. The environmental health practitioners (EHP) educated residents about stress, heat and keeping hydrated.

Members of the team assessed residents’ wardrobes for clothes and toiletries, food storeroom, fridge, residents’ medication, building cracks, roofing, paints, kitchen sink, washing machine, waste management, kitchen, and rehabilitation activities. I noticed that the caregivers communicate via WhatsApp about the whereabouts of the team as they move from one house to the other. The team asked the manager if they can come back tomorrow to finish with the work because they were tired and wanted to leave. The manager refused, and said they have a facility meeting. This was to avoid another busy day preparing for the team.

The team provided feedback and recommendations to the manager and administrator. They first applauded the manager for consistency in service training, patient care and controlling residents from smoking. The nurse asked the manager to take some residents for false teeth and eyes evaluation. She recommended that residents should have time to sleep during the day, especially those with diabetes. The OT recommended that caregivers should help residents with orientation by teaching them their names, medication they take and dates. The finance told the manager that they need to spend their money wisely and save some to buy a facility phone and washing machine. He also mentioned that they will have to write a letter to justify that they need to buy the phone and machine as per the licensing requirements. The EHPs asked the manager to do the following: to improve the ceiling, move waste outside the office, to ensure emergency water supply, fix the screw of the locker, repair peeling paint. They also raised concerns about the safety of the residents when they use toilet located outside at night rather than using the staff one. The OT said she assessed the residents, and she will group them according to their level of functioning and provide games for each level and explanations of how to play.

**Box 4: Observations of the NGCT doing an audit at facility 2**

The visit observed was an announced visit by the NGCT team. The team consisted of more than 10 members including the manager, coordinator of the team, a new additional social worker and dietician. The visits started with a meeting at which NGCT coordinator introduced everyone to the facility manager and explained their agenda for today. The NGCT coordinator asked the team to check for areas that need urgent improvement because the facility is asking to divert funds to the licensing certificates. He also explained that diverting funds will also mean a delay in things like renovations and maintenance.

All caregivers were in a navy-blue uniform, with name tags and hair nets. The residents were more smartly dressed than usual. All residents were sitting down; they looked uncomfortable around the team. The dieticians checked everything including food storeroom, kitchen, fridge, types of plates, cutlery, cups used, dressing of the cooks. They were not happy with the plates, and they said they will recommend for an alternative. The district nurse was checking residents’ medication and files. She noticed that the facility nurse did not know residents’ individual diagnosis and their surnames. She was not happy with the filling system. She told the facility nurse to implement changes and be vocal at the facility about clinical record keeping. She recommended new ways of reporting that will make it easy to read and understand. The EHPs raised concerns about residents’ toilets with cracks, dormitory floors, building paints, roofing cracks, and backup water.

Later the NGO nurse, assistant nurse and district nurse were working together to assess residents’ blood pressure and pulse. The dieticians were checking the residents’ BMI. They were a few residents that the team raised concerned about them being overweight. The OT worked together with the caregivers to assess residents’ level of functioning individually by asking questions like “today’s date, their name, surname”. The OT was concerned that the residents are not oriented because they didn’t know the date including high functioning ones.

During lunch, the dieticians went to the kitchen to observe how the resident cook dishes for the residents, and how residents wash their hands before eating. The dietician mentioned that they should have a sink outside for the residents to wash their hands rather than using a dish. While they were serving food for the residents, the facility administrator called the team to the board room for lunch prepared by the facility. Some residents and caregivers were complaining that they don’t get the nice food provided for the team.

After lunch, the dieticians, OT and nurse went to continue with the assessments of residents. Other team members were asking them to finish it off tomorrow because they were tired. The team left at 3pm and agreed to continue the following day.

### Perceived benefits of regulation

All the compliance team members said that regulating the residential facilities is important to ensure that the services are aligned with the licensing guidelines and promote quality of care: “…*the NGC team is here to see whether NGOs treat the residents accordingly, and they comply with the requirements as stipulated in the licensing guidelines…” (NGCT member 1).* Most family members appreciated the team for inspecting the residential facilities. They described how inspection will help monitor service delivery. For example, this family member stated: *“Well, I think it is good… Life Esidimeni was as a result of NGOs not being checked as to what is the state of those places. So, I think that is something essential because they are people that cannot look after themselves” (Facility3, Family member 1).*

The compliance team mentioned that they should monitor the residential facilities because some of them are making a business rather than taking care of the residents*: “it is a business…the grant money is used to take care of the managers and the board, and not the [residents]” (NGCT member 1).* Facility managers understood the importance of licensing and felt that some residential facilities cannot be trusted with how they provide care to the residents. However, one facility manager hoped that the team would understand that passion for providing care is as important as infrastructure: *“...Unfortunately, the good homes get painted with the same brush as the bad homes. I’m hoping that they will take the NGO as an individual. We are responsible for human life., I don’t have much affiliation with other [facility] managers, I don’t know if they’re passionate about it as we are. When my patients are sick, I worry…” (Facility3, Manager).* Another manager was concerned the NGCT expected all services to be the same, yet the residential facilities do not have similar resources to provide care: *“they can’t paint us all in one brush [*treating facilities as being the same*], what you see in my NGO, it is not what you will find in other NGOs” (Facility1 manager).*

When asked about their role, all team members describe it as it is written in the licensing guidelines. For example, NGCT member 2 stated: *“We audit [NGOs] for compliance, assess their homes, assess the services they render and assist them with the licensing requirements. We support them, we educate them, provide training to the care workers and the managers. We sit in adjudication where we present the NGO [to GDOH] after auditing and assessments. After we discuss whether the NGO meets the requirements for licensing or not” (NGCT member 2).* The other team member shared: “*When we audit the NGO, we come and identify if new or the same problems still exist, go back to the drawing board and find better or simple ways [to address them] according to the level of the NGO because now whether we like it or not, NGOs are not of the same level. From there, we support them accordingly” (NGCT member 1).*

The team lacked sufficient resources to conduct the inspections and support NGOs. The compliance team described how they do not have work telephones to assist NGOs to trace family members nor laptops to help them keep records and write reports. The team tries to work with the limited resources of the NGOs: *“When I recommend activities, I must make sure that the activities that I recommend are low cost” (NGCT member 9).* We observed the compliance team nurse asking to use the NGO’s BP monitor machine, glucometer, cotton balls and body weight scale to assess residents’ health: “*Usually we do not have resources, we use what we get at the NGO” (NGCT member 9).*

However, the managers felt the team could support them more by providing professional mental health staff*: “If they can meet us halfway and give us an OT… or a social worker once a week. She could go to another organization the other week…” (Facility1 manager).* One manager described how the governance team could provide training, co-ordinate and link NGOs with services: *“the Department needs to step up, if you want professional training for care workers, provide us with training… the Department of Health could speak to the municipality. They have the contractors, they have the resources, they have the social workers, help us” (Facility3, Manager)*.

The managers appreciated the teams’ support with regards to the municipality certificates: *“Actually, they are trying to help because they are also engaging with the municipality to speed up the process” (Facility2, manager).* They also appreciated the teams’ help when a resident dies: *“I want to give thanks to NGC team manager and his assistant. When we have funerals, they come to see the [residents] because some of them [residents], are so shocked that they’re going to die. So, they comfort us” (Facility1 manager).*

### Suitability of licensing guidelines to residential services

When asked about their understanding of residential care, the NGCT described it as a home. For example, this team member stated*: “it is a home away from home…a home that is well equipped to take care of the patient, wholeheartedly, holistically…Besides the safety, the nutrition and everything, mainly where they will receive love and care”* (NGCT member 3). The other team member described it as an institution: *“it is a non-government institution for people that suffer from serious mental illness that cannot be in the community. Still need medication and be stabilised and be rehabilitated”* (NGCT member 2).

The facility managers felt the licensing regulations were for a hospital setting rather than a residential service: “*I think my family’s objective was to create a [residential] environment. A lot of the recommendations coming from the department are very rigid. It’s almost a hospital recommendation, you know, when to eat, when to take the tablets, how many hours in OT…” (Facility3, Manager).* The other manager stated “*All the things they [NGCT] want were at hospital level, we are not hospital. We give [users] a home away from home” (Facility1, Manager).*

During inspection, SM observed the NGCT’s dietician telling the facility manager to buy plastic plates that are similar to the ones used at Baragwanath [hospital]. The manager had a problem with plastic plates: “*We bought those plates, and when you put them in the microwave, they crack or when you put hot food, they crack. You cannot compare us with what is happening in Baragwanath, this is not a hospital, it is a [residential facility]” (Facility2, Manager).* SM also observed the team’s occupational therapist telling the facility manager that she will design a timetable of activities for the residents. The manager raised concerns about having a schedule of events in a residential facility. She described how residents get anxious when they are forced to do activities. “*The minute something feels too forced, you create an anxiety in them…If it’s forced, they will jump the wall because they don’t feel at home anymore. The minute you say listen “you will participate in an eight-hour program… they start thinking there must be something better”. (Facility3, Manager).*

### Challenges in complying with the guidelines

The facility managers mentioned that they were not adequately consulted during the gazetting of the regulations, yet they are more experienced in delivering residential services than the compliance team: *“We know what works, it’s not something you’re going to read in a book. You understand, we take the recommendations, and we take the instruction and recommendations from the department, but we know what works... We’ve…grown over the years (Facility3, Manager).* Another facility manager described how the Department [of Health] is making decisions on their behalf without being at the ground level: *“I think because we don’t have a link between the Department and us, the Department is making decisions, but they’re not on our ground level. The government is making decision of doing policies, acts but there is no one from the NGO with experience making decisions on our behalf” (Facility1, manager).*

The team was aware that the subsidy from the government is not enough for the residential facilities to afford the licensing requirements: *“The funding sometimes is not enough to meet all these requirements cause the list is long …with the little money that they are receiving, it won’t be easy. We will end up being stuck with non-compliances…” (NGCT member 8).* The managers highlighted that the licensing requirements are costly, and they do not have enough funds: *“…Most of the requirements need money. And the government is just subsidizing us. The subsidy is [not enough] (Facility1 manager).* This manager further stated that the government gives them a breakdown of how they should spend the subsidy money. The breakdown of the subsidy money is not aligned with their needs*: “It is challenging because the department is already giving you a budget breakdown for the money. They said human resource 60% but you cannot give 60% to human resource with this kind of service. They said 35% service, how can you have 35% when you are dealing with people that break windows any day when they relapse…” (Facility1 manager).* To align the subsidy breakdown with the facility needs, the managers try to negotiate with the NGCT for them to direct some funds to what takes priority to meet the licensing requirements: *“Should you say on rehab equipment, we’re okay. I’m going to reduce that to zero…because maintenance at this stage takes priority. If you push too hard, they say no, correct it, we do not agree to 10,000 maintenance a month... They don’t know what kind of maintenance we are talking about. I mean…when engineers pull in here for renovations…for the CoCs [certificate of compliance]. We’re looking at half a million” (Facility3, Manager).*

The residential facilities have been operating before the introduction of the licensing guidelines. It is challenging for the facilities to adapt to delivering services based on the guidelines: *“some of the requirements are very difficult for the NGOs to comply with. Reason being that the guideline came while NGOs are already in existence. Consultation was minimal. It’s something that was brought to us from national [government] after the Life Esidimeni [tragedy] to try and correct the mistake…” (NGCT member 1)*. As a result, the compliance team is left with the decision to support the licensing of the NGOs even if they do not comply with the regulations because there is a shortage of residential facilities to provide care: *“we must be patient with the NGOs to meet the requirements because there are residents in the NGOs, and we cannot take them anywhere, even if we highlight that the NGO doesn’t fully comply with what we require. We don’t have space; we don’t have accommodation for these people. So we still license the NGO…” (NGCT member 1).*

As specified in the licensing guidelines [Box 3] the residential facilities need to have a number of compliance certificates. Each certificate has certain standards that the residential facility should meet. For example, the compliance team described what the NGO needs to get an acceptability certificate: *“For the municipality to give them the certificate [of acceptability], they must have a handwash basin in the kitchen…and that sink is expensive…” (NGCT member 7).* The facility manager described some of the steps involved when applying for a zoning certificate from the municipality: *“The zoning certificate…from the municipality it’s very expensive... They come and check how is the building and everything before you get the certificate. It’s steps before you get it…” (Facility2, manager).*

However, team members expressed frustration that some facilities do not implement the simpler recommendations: “S*ome of the NGOs, if you do a menu plan for them, when you go there… they’re following their own menu that you didn’t even advise on” (NGCT member 10).* Another team member described how the residential staff does not always follow her recommendations: “*sometimes even though you explain the importance of doing all these things with the [residents], the staff do not do what you’ve recommended…I know it is difficult for them, I know it is a lot of work but I feel like it is boring when you don’t see the results just because the person that was supposed to do what you recommended didn’t do it” (NGCT member 9).*

All residential facility staff members raised concerns about the delays in the payment of government subsidy, which in turn affects when their salaries are paid. For example, a caregiver stated: *“We don’t have a pay date here, it just comes in, you see now the month ended, we could be paid next week, at times maybe on the 15th” (Facility1, Caregiver 4).* This means that when they do not have transport money, they do not go to work. One caregiver stated: *“I stay for about 2 days at home and say I will wait for pay day not knowing when that will be. I will not go around creating debts for myself [borrowing money to pay for transport]” (Facility3, Caregiver 3).*

### The perceptions of residential staff and residents of the NGCT

The facility staff described how they are not always honest to the compliance team during inspection: *“Even to the government we do lie, we should say this[machine] works, knowing very well, that it doesn’t work. When they [compliance team] come, we put nice food on top of the table… and they fill [storeroom with food]” (Facility3, Caregiver 4).* The other facility staff member described how she avoids lying to the compliance team: “*With me when they come, I just walk away and hang clothes outside because I know they [management] say we have to lie and say the machine is working, while I am the one using it and it is not working…At times they are stricter, they can say I must plug it, what will I say…” (Facility2, Laundry lady).*

Most residential staff members shared their experience of how they feel unappreciated by the NGCT during inspections. One residential staff member described how the district team does not care about them: *“…They don’t care to sit down with us private and ask us how it is, how do you feel, what they care about is to check how we clean and how [residents] eat.” (Facility1, Caregiver 5).* The other staff member stated: *“There is nothing the government talks to us about as care workers. The government doesn’t care about us, they care about the management” (Residential3, Caregiver 4).* Some of the staff members shared how the NGCT should care more about them because they are the ones providing care to the residents: *“…the government, doesn’t care about the one taking care of the patients. The government doesn’t know that there are things that I am not happy about with the way patients are being treated, and they can only hear about that from me. I also play the part of being a parent to this person. He forgets that the managers don’t spend as much time as I spend with the patient. A week can end without patients seeing the manager, but with me, I am always here every day. (Facility3, Caregiver 4).*

Residents shared their experiences of changes that resulted from the NGCT recommendations: *“At first, they were giving us the [tobacco to smoke], like in the morning and evening when we are going to sleep. The compliance team said that we should not smoke, and they stopped giving us...We asked to see the supervisor to ask when the cigarette is coming. We know that people that have psych [mental illness] should smoke cigarette to calm down the nerves. He said no he will go ask from the [compliance] team when they are going to bring the cigarettes. Till today, he is not back to us, and it’s been a while” (Facility2, Resident 8)*. The other resident stated: *“There is nothing we can do here. Everything here is managed by the government [compliance team] …” (Facility2, Resident 10).* The other resident shared his views about activities recommended by the district team*: “It is the government in charge. They said that we should do those things, but the handiwork are for kids” (Facility2- Resident 6).* The other residents had this to say about the activities: *“I am no longer happy about them [activities] you see these beads, they can say undo them and do it again, because there is nothing that you can do, you will undo them, like doing the same thing, doesn’t that cause you stress though…every day” (Facility1, Resident 5).*

Some of the residents described how the NGCT recommended that the facility manager should cut down their grant: *“At first, we received the [disability grant] R610, the [manager] reduced it to three hundred because the government [compliance team] said that we use it to buy junk food and cigarettes. So, [the manager would] rather take it and do maintenance” (Facility3- Resident 7)*. Residential facility 3 allowed the residents to have romantic relationships, and some residents were married. However, they were not allowed to stay together as a couple. And when we asked one married resident why she was not staying with her husband she said: *“I don’t understand it, they are saying the [NGCT] won’t allow us to stay together because we are going to have sex and kids. If I have got the implant, why can’t I have sex with my husband” (Facility3-Resident 12)*. The other resident used to stay with his wife. However, the NGCT was against that because they were staying in a shack which is against the licensing guidelines. The resident stated: *“We were staying together until the government said no, you cannot stay together on the property...They condemned the place because the floor was busy collapsing. She went to women’s dorm, and I went to men’s dorm…” (Facility3- Resident 11).*

When SM observed the compliance team assessing the whole facility, the residents’ looked scared of the team. The facility manager explained that *“they feel their home is at risk” (Facility3, manager).* The facility manager described how they conduct inspections: “*Could you imagine you live in your house, and you’ve got your family there and you carry on with daily living, an official comes to walk through your house, check your groceries, check your bedding… how much do you smoke, how much do you eat… checking their toiletry bags…doing a physical assessment” (Facility3, manager).*

The residents shared their experiences of how inspection makes them feel uncomfortable: *“I’m afraid [when inspection people come] because I know they can open my cupboards, they can go into my dresser. They can do whatever they want to do, ask me any question. I’m afraid like what did we do wrong. I am threatened by them” (Facility3, Resident 1).* The other resident described how he feels about the inspection day: *“I don’t really enjoy it [inspection] because now we must stand to attention for them. The place has to be perfect. I am happy when it’s normal. The place gets cleaned every day so why we should go the extra mile, and we must be hidden away in the OTs when they come... I am mentally ill right. That’s what it is known, it is a mental illness. So, why must they treat me like I have mental illness” (Facility3, Resident 2).*

## Discussion

Family members and managers understood the importance of regulating residential care facilities to improve the quality of care. Facility managers felt they were not consulted during implementation of the regulations, yet they have long experience of providing residential care. Facility managers reported that some of the licensing requirements were appropriate for hospitals but were not relevant for residential care. Managers, staff and residents raised concerns about the intrusiveness and lack of flexibility in the inspection process. The facility staff felt neglected and undervalued during inspections while residents felt their lives were controlled by the NGCT. The team tried to help the NGOs meet the licensing requirements but found it challenging to provide support within their own limited resources. NGOs struggled with meeting licensing requirements, mainly due to shortage of funding. The team is left with a decision to license facilities even though they do not meet requirements because there is a shortage of residential care facilities.

The idea of community-based service entails non-coercive practices, the promotion of human rights and person-centred care. The services should aim to help users develop practical living skills and promote independence [25]. Independent living does not mean being self-sufficient [26]. It means that people with disability should have access to support designed in a way that allow them to make choices and take control over their lives [26]. Our study found evidence that the licensing guidelines obliged the NGCT to recommend structured living for the residents of care facilities. This happens when the residential facilities are highly supervised and regulated, with limited autonomy over daily activities of living [27]. A structured life in supported residential services is an institutional model of care [28] rather than person-centred care [29]. This means that residents are not integrated with the community, lack autonomy and liberty [30].

Although there was a public call to comment on the licensing guidelines in 2022, the managers that participated in our study were not aware of this call hence they felt like they were not consulted about the implementation of the regulations. A systematic narrative review about standards in regulating quality of adult community health and social care suggests that it is important to involve the service providers when designing and implementing regulations [31,32]. This enables service providers to understand their task, improve self-governance and provides feedback regarding the regulation process [31,32]. A study from England found evidence that the service users make a significant contribution to the regulation of social care providers by sharing their experiences [33]. Service users helped inspectors to incorporate multiple perspectives on care [33]. The inspectors need to be more aware of their dual role of providing support and reporting on problems that they identified during inspection.

Our study found that the dual role of supporting and inspecting these facilities created tensions for the NGCT. There are studies that have explored the downside of dual role in health care setting [34,35]. The studies found that most health care workers with dual roles face challenges in balancing their responsibilities. Dual role usually leads to service providers experiencing identity conflicts because they struggle to transition from one role to another. Our data suggests that the NGCT may benefit from training focussed on enabling them to balance their dual role of inspecting and supporting the facilities. Alternatively, there should be a separation of duties between the provincial health authorities such as the directorate of mental health and the NGCT district health personnel. The NGCT could focus on supporting the residential facilities to meet the licensing requirements while the provincial health authorities inspect and recommend facilities for licensing.

The NGO facility managers raised concerns about the hospital like requirements for licensing. This suggests that there is a need to clarify the purpose of the residential facilities, whether they should be considered as health establishments which admits patients for medical needs or social care facilities intended to support community integration and improve quality of life among PLSMI. More consideration is therefore needed as to the respective roles of the Department of Social Development and Department of Health.

## Limitations

As far as we are aware, this is the first study to explore the views of service users and providers about the process of licensing and regulating mental health residential homes in South Africa. We used both interviews and observations to collect data which allowed us to obtain multiple perspectives and triangulate data. We asked all the participants about their experience of the licensing regulations. However, most NGO staff, residents and families were unable to provide detailed data about the process of licensing and regulating NGOs as they have limited contact with the NGO governance and compliance team. We had limited information from the NGCT about their experience in supporting and inspecting NGOs. We only selected one NGC team and 3 NGOs as our case study which limits the generalizability of the study findings. We also relied on participant self-report and observations which might lead to bias.

## Recommendations for policy, research and practice

The study highlights the need to further explore the following:

- The appropriateness of the licensing guidelines and the purpose of residential facilities, including whether they should be considered as health establishments or social care facilities.
- If they are health establishments, there is a need to assess the desired service and risk related implications of not complying with certain licensing guidelines. For example, if a residential facility cannot afford the municipality certificates, does it have an impact on the personal well-being of the residents.
- The dual role of the NGCT in terms of supporting the NGOs to meet licensing requirements and inspecting them to assess compliance with the requirements.
- Involvement of the residential staff and residents in decision making to improve the quality of the rehabilitative environment.

## Conclusions

The experiences of service users and providers highlight that the licensing guidelines are not currently fit for the purpose of providing community-based residential services. There is a need to further understand the purpose of residential facilities and whether they should be considered as health establishments or social care facilities. Involvement of service users and providers in mental health care planning may help align the licensing regulations to residential care services that are truly community-based.
